# Aryl Hydrocarbon Receptor–Independent Toxicity of Weathered Crude Oil during Fish Development

**DOI:** 10.1289/ehp.8230

**Published:** 2005-08-10

**Authors:** John P. Incardona, Mark G. Carls, Hiroki Teraoka, Catherine A. Sloan, Tracy K. Collier, Nathaniel L. Scholz

**Affiliations:** 1 Ecotoxicology and Environmental Fish Health Program, Environmental Conservation Division, Northwest Fisheries Science Center, National Oceanic and Atmospheric Administration, Seattle, Washington, USA; 2 Auke Bay Laboratory, Alaska Fisheries Science Center, National Oceanic and Atmospheric Administration, Juneau, Alaska, USA; 3 Department of Toxicology, School of Veterinary Medicine, Rakuno Gakuen University, Ebetsu, Japan; 4 Environmental Assessment Program, Environmental Conservation Division, Northwest Fisheries Science Center, National Oceanic and Atmospheric Administration, Seattle, Washington, USA

**Keywords:** cardiovascular function, fish development, non-point source pollution, oil spill

## Abstract

Polycyclic aromatic hydrocarbons (PAHs), derived largely from fossil fuels and their combustion, are pervasive contaminants in rivers, lakes, and nearshore marine habitats. Studies after the *Exxon Valdez* oil spill demonstrated that fish embryos exposed to low levels of PAHs in weathered crude oil develop a syndrome of edema and craniofacial and body axis defects. Although mechanisms leading to these defects are poorly understood, it is widely held that PAH toxicity is linked to aryl hydrocarbon receptor (AhR) binding and cytochrome P450 1A (CYP1A) induction. Using zebrafish embryos, we show that the weathered crude oil syndrome is distinct from the well-characterized AhR-dependent effects of dioxin toxicity. Blockade of AhR pathway components with antisense morpholino oligonucleotides demonstrated that the key developmental defects induced by weathered crude oil exposure are mediated by low-molecular-weight tricyclic PAHs through AhR-independent disruption of cardiovascular function and morphogenesis. These findings have multiple implications for the assessment of PAH impacts on coastal habitats.

Every recent assessment of coastal habitats worldwide, from tropical reefs to temperate estuaries, has cited land-based pollution or runoff as a major threat to aquatic ecosystem health (Fabricius 2005; Li and Daler 2004; Pew Oceans Commission 2003; U.S. Commission on Ocean Policy 2004). As pervasive components of runoff from impervious surfaces, polycyclic aromatic hydrocarbons (PAHs) are a part of this problem, and there is very little understanding of their biologic impacts on aquatic resources. Because of urbanization and increased heavy vehicle use, storm water runoff and atmospheric deposition are now the largest sources of aquatic PAH contamination (Li and Daler 2004; Lima et al. 2002; National Research Council 2003; Van Metre and Mahler 2003; Van Metre et al. 2000). An understanding of the effects of PAHs on aquatic organisms is essential to understanding fully the impacts of urbanization and non-point source pollution on coastal habitats.

On a smaller scale, oil spills have provided a more conspicuous view of the impacts of PAH pollution on aquatic resources. Hydrocarbons from oil spills can persist in nearshore sediments for decades or longer and have long-term effects on aquatic ecosystems (Peterson et al. 2003; Reddy et al. 2002; Short et al. 2004). The deleterious effects of PAHs on fish early-life stages were investigated extensively after the 1989 *Exxon Valdez* oil spill in Prince William Sound, Alaska, which contaminated nearshore and intertidal spawning grounds for Pacific herring (*Clupea pallasi*) and pink salmon (*Oncorhynchus gorbuscha*) with Alaska North Slope (ANS) crude oil. Field and laboratory studies in these species and others demonstrated a common syndrome of oil-induced embryolarval toxicity that occurs in a range of teleosts, including marine, freshwater, temperate, and tropical species (Carls et al. 1999; Couillard 2002; Heintz et al. 1999; Marty et al. 1997; Pollino and Holdway 2002). This was characterized by pericardial and yolk sac edema, jaw reductions, and curvature of the body axis. Increased weathering of crude oil enriches the fraction of tricyclic PAHs and their alkylated homologs and increases the frequency of malformations (Carls et al. 1999; Heintz et al. 1999). Additionally, delayed mortality also occurred in the absence of external malformations, as indicated by the reduced oceanic survival of pink salmon exposed to weathered crude oil as embryos and released as smolts (Heintz et al. 2000).

The mechanisms leading to PAH-associated malformations and sublethal effects during fish development are unknown. Most PAHs bind the aryl hydrocarbon receptor (AhR), a ligand-activated basic-helix-loop-helix-Per-Arnt-Sim family transcription factor that controls the expression of a battery of genes encoding enzymes that convert PAHs to water-soluble derivates that are excreted, including mixed-function oxygenases such as cytochrome P450 1A (CYP1A) family members (Nebert et al. 2004). Although CYP1A for decades has been the most widely used biomarker for PAH exposure (Whyte et al. 2000), its role as a bioindicator of PAH toxicity has been debated. Genetic analysis in the mouse has led to a dual model in which the AhR pathway mediates both an adaptive response by which xenobiotic compounds are metabolized and detoxified, and a toxic response whereby receptor activation results in negative impacts in the exposed animal (Nebert et al. 2004; Schmidt and Bradfield 1996). Generally, the toxic response occurs with AhR ligands that are poor substrates for CYP enzymes, in particular, halogenated aromatic hydrocarbons such as dioxins and poly-chlorinated biphenyls. Because these ligands are resistant to metabolism, they accumulate in tissues and persistently activate the AhR. On the other hand, PAHs are classically associated with the adaptive response, by which they are eliminated from tissues. Nevertheless, some high-molecular-weight PAHs such as benzo(*a*)pyrene are converted to carcinogenic reactive intermediates by CYP1A (Phillips 1983), and it is widely held that much of the acute toxicity of PAHs is due to oxidative stress and cellular damage arising from CYP1A catalytic activity.

In fish embryos, PAH and dioxin toxicities are usually equated because exposure to potent AhR ligands such as 2,3,7,8-tetrachlorodibenzo-*p*-dioxin (TCDD) induces a super-ficially similar syndrome (Peterson et al. 1993). In zebrafish (*Danio rerio*), a brief exposure to TCDD shortly after fertilization results in the appearance of vascular dysfunction, pericardial and yolk sac edema, and anemia in hatching-stage larvae at 72–96 hr postfertilization (hpf) (Belair et al. 2001; Henry et al. 1997). These effects of TCDD exposure require a functional AhR. Because of genome duplication, many teleosts have two *AhR* genes, *AhR1* and *AhR2* (Hahn 2002). AhR1 protein is most similar in structure to the single mammalian AhRs, whereas AhR2 is divergent. Although TCDD is a ligand for both receptors from several fish species, *AhR2* transcripts are more abundant and widely distributed (Hahn 2002; Karchner et al. 1999), and in zebrafish only AhR2 was found to be a functional receptor for TCDD and other common halogenated AhR ligands (Andreasen et al. 2002). Consistent with these findings, targeted knockdown of AhR2 in zebrafish embryos with antisense morpholino oligonucleotides (MOs) prevented all of the toxic effects of TCDD that occurred within the time frame of morpholino efficacy (Prasch et al. 2003; Teraoka et al. 2003). However, AhR-dependent developmental defects were CYP1A independent. A CYP1A morpholino did not alter dioxin toxicity in zebrafish embryos, implicating other AhR target genes in dioxin pathophysiology (Carney et al. 2004).

A previous analysis showed that micro-molar concentrations of individual tricyclic PAHs representing the homologous series most abundant in weathered crude oil (fluorene, dibenzothiophene, and phenanthrene) caused a syndrome of edema and craniofacial and body axis defects after dose-dependent cardiac dysfunction that was first observed at about 36 hpf (Incardona et al. 2004). These compounds caused cardiac arrhythmias that are characteristic of drugs known to block cardiac K^+^ channels of the human ether-a-go-go–related gene (*HERG*) family (Langheinrich et al. 2003). Among four-ring compounds, chrysene (9 μM), which is enriched in highly weathered crude oil, was nontoxic, whereas pyrene (1–5 μM), which is generally absent from weathered ANS crude oil, induced a syndrome with features similar to TCDD exposure that occurred between 80 and 96 hpf. Here, we show that *a*) both toxic and non-toxic PAHs induce CYP1A in zebrafish embryos, acting tissue specifically through AhR1 and AhR2; *b*) pyrene toxicity is AhR dependent, whereas tricyclic PAH toxicity is not; *c*) weathered ANS crude oil causes a syndrome in zebrafish embryos that is both clearly distinct from TCDD toxicity and consistent with cardiac dysfunction expected from the most abundant tricyclic compounds; and *d* ) the cardiovascular toxicity of weathered crude oil is independent of both AhR1 and AhR2. Rather than mediating the embryo-larval toxicity of weathered crude oil, the AhR pathway confers a measure of protection against the pathophysiologic effects of tricyclic PAHs on the developing fish heart.

## Materials and Methods

### Chemicals.

Dibenzothiophene (> 99%), phenanthrene (> 99.5%), pyrene (> 99%), and chrysene (98%) were obtained from Sigma-Aldrich (St. Louis, MO, USA). Stock PAH solutions were made in dimethyl sulfoxide (DMSO; tissue culture grade; Sigma-Aldrich) at 10 mg/mL, except chrysene (1 mg/mL). DMSO was ≤0.1% in exposure solutions.

### Zebrafish exposures.

Wild-type AB strain zebrafish were maintained and fertilized eggs processed as previously described (Incardona et al. 2004). Fish were treated humanely and anesthetized when necessary. Exposures to individual model PAH compounds were carried out in plastic six-well plates (15–25 embryos in 3 mL) with a static renewal protocol at 28.5°C as described previously (Incardona et al. 2004). All exposures used doses that were above the solubility of the compounds and that, for toxic PAHs, produced effects in 100% of the embryos (Incardona et al. 2004). Each experiment was replicated at least three times. For crude oil exposures, embryos were incubated statically in a water-accommodated fraction (WAF) of crude oil, or in the continuously flowing effluent from a gravel column. The WAF was prepared by an overnight high-energy spin of 50 mL ANS crude [partially weathered by heating to 70°C until 20% reduction in mass (Marty et al. 1997)] in 30 L zebrafish system water using a large fiberglass tank and a paint mixer fixed to a fan motor. The WAF contained an estimated 2.8 mg/L total PAH (initial) but has the disadvantage of a high proportion of other oil components such as alkanes because a large fraction is present in particulate or colloidal form. WAF was diluted with system water to 1:2, 1:5, 1:10, and 1:100, and static exposures were performed in 30 mm glass Petri dishes (25 embryos in 4 mL) in an incubator at 28.5°C.

We used oiled gravel columns to achieve a more environmentally relevant exposure. System water was passed by gravity through gravel (4–6 mm grain diameter) coated with partially weathered ANS crude oil (6.0 g oil/kg gravel) to model conditions in oiled intertidal substrate as described previously (Marty et al. 1997; Short and Heintz 1997). Controls were similarly incubated in water passed through clean gravel. Dosing columns were 2-L glass beakers filled with 1.3 kg rock, with water flow directed to the bottom through 6 mm glass tubes. Columns were placed in glass baking dishes set at a slight angle (~ 6°); effluent overflowed from the tops of the columns, filling the baking dishes as a reservoir for exposing embryos in replicate open glass 30 mm Petri dishes (*n* = 4–5; ~ 25–50 embryos/dish). Temperature was maintained with submersible aquarium heaters. Column flow was initiated 1 day before embryo exposure to further weather the oil and remove any particulates. Exposures were started at 4–8 hpf. Temperature (nearest 0.5°C) and flow rate (nearest milliliter) were recorded as often as hourly during the day and at one or two time points during the night [Supplemental Table 1; Supplemental Material available online (http://ehp.niehs.nih.gov/docs/2005/8230/supplement.pdf)]. Although embryos developed more slowly at the experimental temperatures, they were staged according to the published standard series (Kimmel et al. 1995), and all developmental times are reported as hpf at standard temperature (28.5°C). We performed six oiled gravel experiments; two included uninjected embryos only (*n* > 300), three included AhR2 morphants (*n* > 200), two included AhR1/AhR2 double morphants (*n* = 145), and one included CYP1A morphants (*n* = 87). Standard statistical analyses were carried out with Microsoft Excel 2004 for Mac (Microsoft Corporation, Redmond, WA, USA).

### Morpholino injections.

*AhR1* cDNA and genomic sequences are available as GenBank AF258854 (GenBank 2005) and Ensembl ENSDARG00000020046 (Ensembl 2005), respectively, and *AhR2* cDNA sequence as GenBank AF063446 (GenBank 2005). All morpholinos were synthesized by GeneTools (Philomath, OR, USA) and are listed below (mismatch nucleotides in control morpholinos are indicated by lowercase letters). The translation-blocking MOs targeting zfAHR2 (5′-TGTACCGATACCCGCCGACATGGTT-3′ for zfAHR2-MO, 5′-TGaACCcATACCCGCCGtCATcGTT-3′ for the negative control 4Mis-AHR2-MO) and zfCYP1A (5′-TGGATACTTTCCAGTTCTCAGCTCT-3′) have been described previously (Teraoka et al. 2003). Splice-blocking morpholinos for zfAhR1 were designed to target the exon 2/intron 2 splice donor site (5′-CTTTTGAAGTGACTTTTGGCCCGCA-3′ for E2I2-MO, 5′-CTTTTcAAcTGAgTTTTGcCCCcCA-3′ for 5Mis-E2I2-MO) and the intron 2/exon 3 splice acceptor site (5′-GTTCAGGGTTACTGCAAAAGAAAT-3′ for I2E3-MO). Morpholinos were injected at the 1–4 cell stage (0.25–1 hpf) as previously described (Incardona et al. 2004; Teraoka et al. 2003), and embryos were allowed to recover in system water at 28.5°C to 50% epiboly (5–6 hr) before use in exposure studies. AhR1 morpholinos were labeled with fluorescein, whereas AhR2 and CYP1A morpholinos were not. For injections involving AhR1, embryos were selected on an epifluorescent stereoscope based on fluorescence intensity and an even distribution in blastomeres.

### Imaging of live embryos/larvae, immunofluorescence, and confocal microscopy.

Digital still micrographs were obtained and video-microscopy of live embryos and larvae performed as described previously (Incardona et al. 2004). Antibodies used were monoclonal 1-12-3 against fish CYP1A (Park et al. 1986), anti-myosin heavy chain monoclonal MF20 (Developmental Studies Hybridoma Bank, University of Iowa, Iowa City, IA, USA) (Bader et al. 1982), and anti-atrial myosin heavy chain monoclonal S46 (Berdougo et al. 2003). For CYP1A immunofluorescence, embryos were fixed overnight in 4% phosphate-buffered paraformaldehyde, and MF20/S46 immunofluorescence was assessed in embryos fixed in either paraformaldehyde or methanol plus 10% DMSO. Processing for immunofluorescence was carried out as described previously (Incardona et al. 2004). Secondary antibodies were AlexaFluor488-conjugated goat anti-mouse IgG1 (S46) and AlexaFluor568-conjugated goat anti-mouse IgG2b (MF20), both from Molecular Probes (Eugene, OR, USA). Immunolabeled embryos were mounted in glycerol or 3% methylcellulose and imaged using a Zeiss LSM 5 Pascal confocal system with Ar and HeNe lasers (Carl Zeiss Advanced Imaging Microscopy, Jena, Germany). For semiquantitative comparisons, treated or control embryos were marked by tail clipping and mixed for antibody labeling, mounted together, and imaged with identical settings.

### PAH analysis.

Water samples (200 mL) were collected and stored in brown glass bottles with 20 mL dichloromethane at 4°C for up to 7 days before extraction. After addition of deuterated internal standards, samples were extracted twice with dichloromethane (25 mL each time) for 2 min each with 2 min separation times using 1-L separatory funnels. For quality assurance, a known mixture of PAHs was added to 200 mL zebrafish system water (spiked blank) and extracted. The extracts were processed and hydrocarbons analyzed by gas chromatography–mass spectrometry using selected ion monitoring as previously described (Sloan et al. 2004). The accuracy of the hydrocarbon analyses was estimated by recoveries from the spiked blank, which ranged from 69 to 111% for 17 different PAHs (naphthalene, 102%; fluorene, 85%; dibenzothiophene, not determined; phenanthrene, 98%; chrysene, 83%). Total PAH concentrations were calculated by summing concentrations of individual PAHs. Relative PAH concentrations were calculated as the ratio of PAH concentration to the total PAH concentration.

## Results

### Differential activation of AhR1 and AhR2 by model PAHs.

We first examined the relationship between CYP1A induction via the AhR and the developmental toxicity of individual PAH compounds [structures shown in Supplemental Figure 1; Supplemental Material available online (http://ehp.niehs.nih.gov/docs/2005/8230/supplement.pdf)]. To determine the roles of the two zebrafish AhRs, we used the previously described (Prasch et al. 2003; Teraoka et al. 2003) translation-blocking AhR2 morpholino (AhR2-MO), and designed AhR1 splice-blocking morpholinos targeting either the exon 2/intron 2 junction or the intron 2/exon 3 junction (Supplemental Figure 1; Supplemental Material available online (http://ehp.niehs.nih.gov/docs/2005/8230/supplement.pdf)]. Morpholinos designed to block splicing or translation have similar efficacy (Draper et al. 2001). Coinjection of AhR2-MO and AhR1 exon 2/intron 2 morpholino failed to produce normal embryos, so all studies used intron 2/exon 3 morpholino (AhR1-MO). Nonfunctional control morpholinos included the AhR2 sequence with a four-base mismatch (AhR2-MIS) and the AhR1 exon 2/intron 2 sequence with a five-base mismatch (AhR1-MIS).

**Figure 1:**
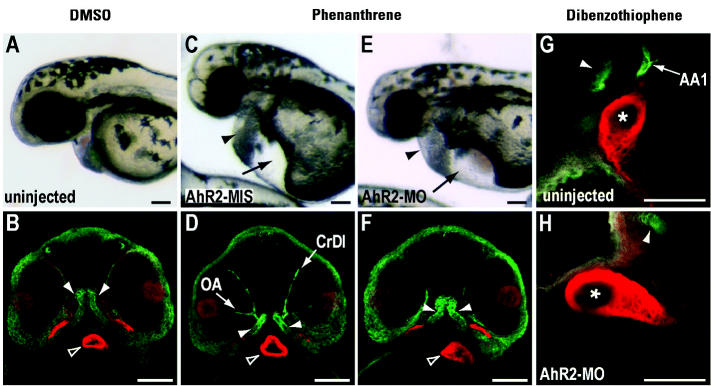
AhR2 knockdown prevents CYP1A induction by phenanthrene and dibenzothiophene, but not cardiac dysfunction. (*A*–*F*) Lateral light microscopic views of live embryos at 48 hpf (anterior at left) are paired with corresponding ventral confocal images (anterior at top) of CYP1A (green) and myosin heavy chain (red) immunofluorescence. (*A, B*) Embryo exposed to solvent (DMSO). (*C, D*) AhR2-MIS–injected embryo exposed to 28 μM phenanthrene. (*E, F*) AhR2 morphant exposed to 28 μM phenanthrene. Black arrowheads and arrows (*C*, *E* ) indicate pericardial and yolk sac edema, respectively. CYP1A immunofluorescence induced by phenanthrene (*D*) in the cranial division of the internal carotid artery (CrDI) and optic artery (OA) was blocked by AhR2-MO injection (*F*). Solid white arrowheads indicate cross-reactive immunofluorescence in the jaw cartilage, and unfilled white arrowheads indicate the ventricular myocardium. (*G*, *H*) Higher magnification confocal images showing CYP1A (green) and myocardial myosin heavy chain (red) immunofluorescence at 48 hpf in embryos with cardiac dysfunction after exposure to 28 μM dibenzothiophene (lateral views with anterior at left). In an uninjected embryo (*G*), the proximal portion of the mandibular arch (AA1, arrow) is CYP1A^+^, whereas the ventricular endothelium is CYP1A^−^ (asterisk). Only cross-reactive signal is seen in the jaw cartilage (arrowhead) in an AhR2 morphant (*H*). Bars = 100 μm (*A*–*F*) and 50 μm (*G, H*).

At nominal concentrations ranging from 10 to 60 μM, phenanthrene or dibenzothiophene causes dose-dependent changes in cardiac rhythm ranging from bradycardia through partial (2:1) to complete atrio-ventricular (AV) conduction block (Incardona et al. 2004). In embryos exposed to approximately 30–60 μM phenanthrene or dibenzothiophene with AV block and edema [Figure 1C, Supplemental Movie 1; Supplemental Material available online (http://ehp.niehs.nih.gov/docs/2005/8230/supplement.pdf)], relatively weak CYP1A immunofluorescence was observed predominantly in vessels most proximal to the heart, including the first aortic arch and the carotid artery (Figure 1D,G). However, CYP1A immunofluorescence was not observed in endothelial cells lining the heart in embryos exposed to either phenanthrene or dibenzothiophene (Figure 1D,G). Injection of AhR2-MO largely blocked CYP1A induction by dibenzothiophene and phenanthrene (Figure 1F,H; Table 1). However, despite this effect on CYP1A induction, AhR2 morphants (i.e., MO injected) were not protected from phenanthrene- or dibenzothiophene-induced cardiac dysfunction (Figure 1E, Table 1). The same types of cardiac arrhythmia were observed in AhR2 morphants exposed to phenanthrene or dibenzothiophene at the same developmental stage as controls [~ 36 hpf; data not shown, Supplemental Movie 1; Supplemental Material available online (http://ehp.niehs.nih.gov/docs/2005/8230/supplement.pdf)]. Although we did not quantify changes in the dose response, AhR2 morphants generally had a higher degree of AV block than did controls at a given phenanthrene or dibenzothiophene concentration, consistent with a protective effect of CYP1A induction. Injection of AhR1-MO alone did not prevent CYP1A induction by phenanthrene (data not shown) and, in combination with AhR2-MO, did not prevent tricyclic PAH toxicity (Supplemental Movie 1; Supplemental Material available online (http://ehp.niehs.nih.gov/docs/2005/8230/supplement.pdf)]. Therefore, although the tricyclic PAHs phenanthrene and dibenzothiophene induce CYP1A weakly in some blood vessels through AhR2, the primary toxicity of these PAHs in fish embryos is AhR independent, and their cardiac effects are not associated with AhR activation or CYP1A induction in the endocardium.

**Figure 2:**
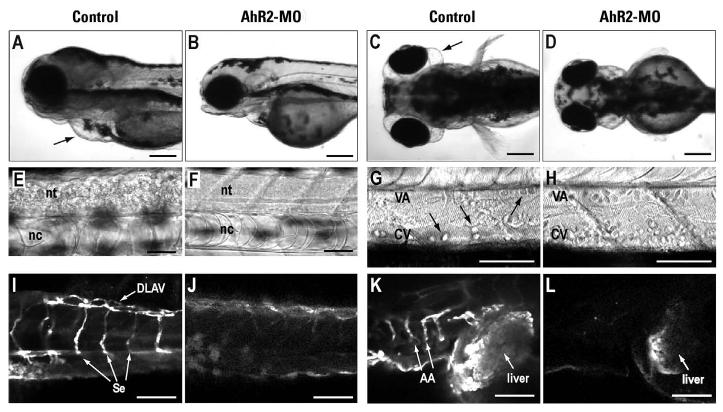
AhR2 morphants are resistant to pyrene toxicity. Control (uninjected or AhR2-MIS injected) and AhR2 morphant embryos were exposed to 5 μM pyrene through 100 hpf. Lateral (*A*, *B*) and dorsal (*C*, *D*) views showing edema (arrows) in uninjected larvae. Higher magnification light micrographs of the trunk region of uninjected (*E*, *G*) and AhR2 morphant (*F*, *H*) larvae showing cell death (*E*, granular appearance) in the neural tube (nt, neural tube; nc, notochord) and a reduction of erythrocytes (*G*, arrows) in the ventral aorta (VA) and caudal vein (CV) of uninjected larvae. (*I–L*) CYP1A immunofluorescence in the trunk (*I*, *J*) and head regions (*K*, *L*) of pyrene-exposed larvae. In AhR2-MIS–injected larvae (*I*, *K*) the vasculature (DLAV, dorsal longitudinal anastomotic vessel; Se, intersegmental vessels; AA, branchial arches) and liver are CYP1A^+^, whereas only weak signal is seen in the liver of the AhR morphant (*J*, *L*). In (E–L,) anterior is to the left and dorsal at top. Bars = 200 μm (*A*–*D*) and 50 μm (*E*–*L*).

In contrast to the tricyclic PAHs, the effects of pyrene during zebrafish development overlap considerably with those previously reported for TCDD exposure. Despite widespread induction of CYP1A throughout the vascular endothelium observed as early as 36 hpf (Table 1 and data not shown), the overt signs of pyrene toxicity do not appear until after 80 hpf (Figure 2). By 100 hpf, uninjected or AhR2-MIS–injected embryos exposed to pyrene showed edema (Figure 2A,C and data not shown), cell death in the neural tube (Figure 2E), and anemia (Figure 2G), as well as CYP1A immunofluorescence in the liver (Figure 2K) and throughout the vascular endothelium of the trunk and head (Figure 2I,K). Most pyrene-exposed larvae die later in the fifth day of development (Table 1). Injection of AhR2-MO largely prevented the morphologic and lethal effects of pyrene exposure (Figure 2B,D,F,H; Table 1) and markedly reduced the levels of CYP1A immunofluorescence assayed at both 48 hpf (Table 1) and 100 hpf (Figure 2J,L). At comparable time points, CYP1A morphants exposed to pyrene generally had defects that were less severe than those of controls. For example, pericardial edema was more severe at 96 hpf in AhR-MIS–injected larvae than in CYP1A morphants, indicated by pericardial cross-sectional areas of 0.034 ± 0.008 mm^2^ and 0.021 ± 002 mm^2^, respectively (*n* = 10 for each, *t*-test *p* < 0.001). However, CYP1A-MO injection did not ultimately protect from pyrene toxicity because all of the defects associated with pyrene exposure (including lethality) appeared in CYP1A morphants 12–18 hr later than in controls, possibly due to loss of MO efficacy at later stages (Table 1). These findings indicate that, like phenanthrene and dibenzothiophene, pyrene selectively activates AhR2, but unlike these tricyclic PAHs, pyrene toxicity is AhR dependent. Moreover, delay of pyrene toxicity in CYP1A morphants suggests the involvement of a toxic CYP1A-derived pyrene metabolite.

**Figure 3:**
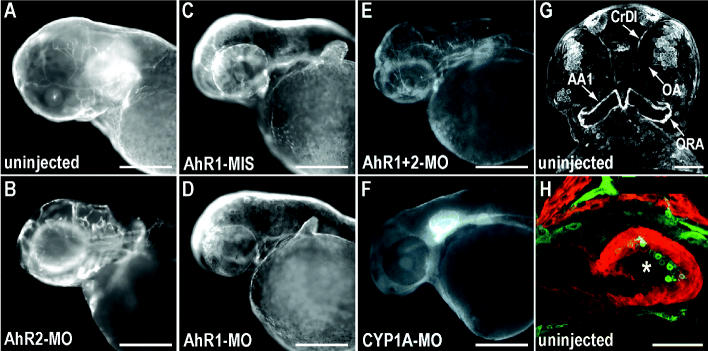
Chrysene induces CYP1A through both AhR1 and AhR2. All images show CYP1A immunofluorescence at 72 hpf (*A*, *B*, *G*, *H*) or 48 hpf (*C*–*F*) after exposure to 9 μM chrysene from 6 hpf. (*A*–*F*) Lateral epifluorescent images with anterior to the left in uninjected (*A*), AhR2 morphant (*B*), AhR1-MIS–injected (*C*), AhR1 morphant (*D*), AhR1/AhR2 double morphant (*E*), and CYP1A morphant (*F* ) embryos. Epidermal CYP1A is seen as punctate fluorescence on the surface of the embryos. Immunofluorescent signal in the otic capsule and jaw cartilage was often observed in unexposed embryos. This signal was resistant to CYP1A morpholino (*F*) and is therefore likely to represent a cross-reactive protein. (*G*, *H*) Confocal images of uninjected chrysene-exposed embryos. (*G*) Three-dimensional confocal projection (180 μm series of optical sections) of CYP1A immunofluorescence, ventral view with anterior at top. Arrows indicate CYP1A^+^ blood vessels; AA1, mandibular arch; CrDI, cranial division of the internal carotid artery; OA, optic artery; ORA, opercular artery. (*H*) Confocal optical section through the cardiac chambers (anterior at top) with CYP1A (green) and myosin heavy chain (red) immunofluorescence. The asterisk (*) indicates CYP1A^+^ endothelial cells lining the ventricle. Bars = 250 μm (*A*–*F*) and 50 μm (*G, H*).

A trivial explanation for the absence of embryonic toxicity for some compounds [e.g. chrysene (Incardona et al. 2004)] could be a lack of tissue uptake during the course of the exposure. For example, because of differences in water solubility of PAHs with different ring arrangements but similar molecular weight (e.g., 0.67 μM pyrene vs. 0.009 μM chrysene), the effective exposure levels would be very different. However, exposure to 9 μM chrysene, which produces no apparent toxic effects, resulted in robust CYP1A immunofluorescence throughout the epidermis and the vascular endothelium (Figure 3A,G). Most cranial (Figure 3G and data not shown) and trunk vessels (data not shown), as well as the endothelial cells lining the cardiac ventricle (Figure 3H), expressed CYP1A after chrysene exposure. Remarkably, injection of AhR2-MO blocked induction of CYP1A in the epidermis by chrysene while leaving the vascular induction intact and generally increased (Figure 3B). In contrast, injection of AhR1-MIS (Figure 3C) or AhR1-MO (Figure 3D) had no effect on CYP1A induction by chrysene, but coinjection of both AhR1-MO and AhR2-MO markedly reduced both epidermal and endothelial CYP1A immunofluorescence (Figure 3E). Injection of CYP1A-MO eliminated virtually all CYP1A immunofluorescence associated with chrysene exposure (Figure 3F). These findings indicate that chrysene can activate both AhR1 and AhR2 in a tissue-specific manner. Although either AhR1 or AhR2 can mediate the vascular induction of CYP1A by chrysene, the epidermal induction of CYP1A by chrysene is AhR2 specific. However, AhR activation and CYP1A induction by chrysene are not associated with any overt developmental toxicity or cardiac dysfunction (despite CYP1A induction in cardiac endothelial cells).

### Embryonic cardiac dysfunction and the weathered crude oil syndrome.

**Figure 4:**
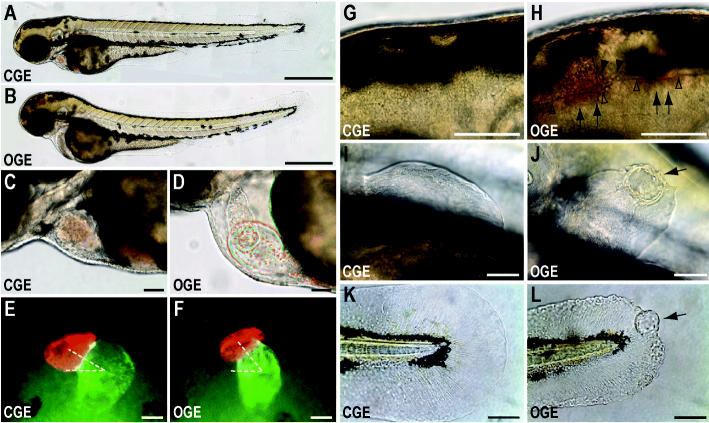
Defects resulting from embryonic exposure to OGE. PAH levels are shown in Table 2 (results for column 1) and Supplemental Figure 2 [Supplemental Material available online (http://ehp.niehs.nih.gov/docs/2005/8230/supplement.pdf)]. (*A, B*) Gross appearance at 64 hpf of CGE-exposed (*A*) or OGE-exposed (*B*) larvae. (*C, D*) Cardiac morphology at 64 hpf in CGE-exposed (*C*) and OGE-exposed (*D*) larvae. (*E, F*) Cardiac chamber–specific immunofluorescence (red, ventricle; green, atrium) in CGE-exposed (*E*) and OGE-exposed (*F*) larvae at 39 hpf; dashed white lines indicate the angles measured to assess looping. (*G, H*) High-magnification views of the midbrain–hindbrain junction in CGE-exposed (*G*) and OGE-exposed (*H*) larvae. Arrows indicate red tinge from extracellular hemoglobin; arrowheads mark extravascular erythrocytes, and the floor of the brain ventricles is marked with unfilled arrowheads. (*I*–*L*) High-magnification views of pectoral (*I*, *J*) and caudal (*K*, *L*) fins in CGE-exposed (*I*, *K*) and OGE-exposed (*J*, *L*) larvae. The finfolds of OGE-exposed larvae have irregular margins and blisters (arrows). Bars = 500 μm (*A, B*) and 50 μm (*C*–*L*).

We exposed zebrafish embryos to ANS crude oil weathered using two methods: generation of a WAF by an overnight high-energy spin of an oil–water mixture and incubation in the effluent from a column loaded with oiled gravel (OGE) or control (clean) gravel (CGE). Although chemical analysis demonstrated that these two methods produced different degrees of weathering [Table 2, Supplemental Figures 2 and 3; Supplemental Material available online (http://ehp.niehs.nih.gov/docs/2005/8230/supplement.pdf)], exposure of embryos to both preparations (WAF exposed and OGE exposed) produced identical types of biologic effects (Figure 4 and data not shown).

In general, the defects in WAF-exposed embryos were more severe, consistent with its higher degree of weathering (i.e., larger tricyclic PAH fraction). By 63 hpf, WAF- or OGE-exposed embryos showed a suite of defects that overlapped considerably with exposure to individual model tricyclic PAHs, but with additional features not observed with the model compounds. Grossly, all embryos exposed to weathered crude oil showed dorsal curvature of the body axis; mild to moderate pericardial edema was seen in OGE-exposed embryos (Figure 4B,D), and yolk sac edema was seen in WAF-exposed embryos (data not shown). In both types of exposures, changes in cardiac function were the earliest observed defects. Mild pericardial edema and reduced blood flow associated with poor cardiac contractility and bradycardia were apparent in WAF-exposed embryos at 33 hpf (data not shown) and by 36–39 hpf in OGE-exposed embryos [Supplemental Movie 2; Supplemental Material available online (http://ehp.niehs.nih.gov/docs/2005/8230/supplement.pdf)]. Usually, the first and mildest sign of cardiac dysfunction was regurgitation of erythrocytes from the atrium into the yolk sac [Supplemental Movie 3; Supplemental Material available online (http://ehp.niehs.nih.gov/docs/2005/8230/supplement.pdf)]. Staining with the chamber-specific antibodies MF20 and S46 demonstrated that subtle delay or disruption of cardiac looping was associated with the earliest observed defects in cardiac function (Figure 4E,F). Cardiac looping was assessed quantitatively by measuring the angle between the cardiac chambers relative to the left–right body axis (Figure 4E,F). CGE-exposed embryos fixed at 39 hpf had a mean inter-chamber angle of 28 ± 5° (*n* = 6), whereas OGE-exposed embryos with weak contractility had a mean interchamber angle of 50 ± 2° (*n* = 7, *t*-test *p* < 0.01). All WAF- or OGE-exposed embryos showed severely abnormal cardiac looping at subsequent stages (typically by 54–64 hpf), and exposures were terminated at 72–80 hpf. The late cardiac morphology typically showed chambers that were stretched along the anterior–posterior axis, with the ventricle stiff and reduced in diameter and the atrium relatively dilated (Figure 4D). Atypical movement was often observed in the wall of the cardiac outflow tract or bulbus arteriosus [Supplemental Movie 4; Supplemental Material available online (http://ehp.niehs.nih.gov/docs/2005/8230/supplement.pdf)]. In most cases, and in particular in WAF-exposed embryos, both cardiac chambers ultimately collapsed into a stringlike structure when embryos were exposed for longer durations (data not shown).

Intracranial hemorrhage and doming of the head due to ventricular edema was very common among embryos exposed to either preparation (85% of OGE-exposed embryos in one experiment, *n* = 66) and most often involved the mesencephalon/third ventricle and hindbrain/fourth ventricle (Figure 4H). Hemorrhage occasionally involved the branchial arches or eye but was never observed in tissues outside the head (data not shown). The sensitive period for intracranial hemorrhage was late during the second day of development to early in the third day; although not observed at 30 hpf, hemorrhage first occurred between 30 and 39 hpf, with the number of affected embryos maximal by 58–63 hpf.

Finfold defects involving all fins and consisting of irregular edges or blisters were also common by 63–72 hpf (94% of OGE-exposed embryos, *n* = 66; Figure 4J,L). No abnormalities were observed in CGE-exposed embryos.

Although we did not observe arrhythmias typical of AV conduction block in OGE-exposed embryos, the total PAH levels were well below the levels at which either phenanthrene or dibenzothiophene alone blocks AV conduction. The key finding is that the complex PAH mixture that comprises weathered crude oil caused early cardiac dysfunction similar to effects of model tricyclic PAHs, rather than a syndrome that arises later during the larval period, such as that associated with pyrene or TCDD exposure. Weathered crude oil also produced additional defects in zebrafish embryos (intracranial hemorrhage and finfold defects) that either are not observed with exposure to individual model PAHs or dioxins, or arise during an earlier developmental stage than AhR-mediated TCDD toxicity.

### A protective role for the AhR pathway.

**Figure 5:**
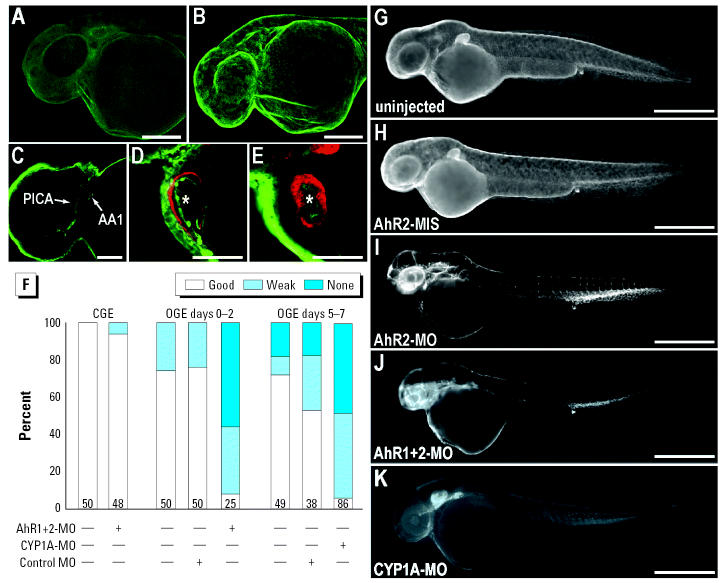
AhR1/AhR2 or CYP1A morphants are more sensitive to weathered crude oil toxicity. (*A*–*E*) Confocal immunofluorescence images of CYP1A (green) and myosin heavy chain marking myocardium (red). CGE-exposed embryos showed no CYP1A immunofluorescence at 39 hpf (*A*), whereas OGE-exposed embryos showed intense immunofluorescence in the epidermis (*B*; 180 μm series of optical sections, vasculature of the head (*C*, optical section; PICA, primitive internal carotid artery; AA1, mandibular arch), and endocardium (asterisks in *D*, *E*) in both the atrium (*D*) and ventricle (*E*). (*F*) Cardiac function (good: strong forward flow; weak: forward flow with atrial regurgitation; or none: erythrocytes pooled in the yolk sac) at 39–40 hpf in CGE- and OGE-exposed embryos that were uninjected, control morpholino injected, AhR1/AhR2 double morphant, or CYP1A morphant [Supplemental Movie 3; Supplemental Material available online (http://ehp.niehs.nih.gov/docs/2005/8230/supplement.pdf)] Bars represent the percentage of embryos in the three classifications (numbers within each bar indicate *n*). The data represent sets of embryos that were exposed sequentially in the effluent of a single pair of columns (control and oiled) run over 8 days. PAH levels are shown in Table 2 (results for column 6) and Supplemental Figure 3 [Supplemental Material available online (http://ehp.niehs.nih.gov/docs/2005/8230/supplement.pdf)]. Because weathering increases with duration of column flow, AhR1/AhR2 double morphants (AhR1+2-MO) and corresponding controls (bars under OGE days 0–2) were exposed to less weathered oil than were the CYP1A morphants and controls (bars under OGE days 5–7; see also Table 2). The increased severity of cardiac dysfunction in control embryos exposed during days 5–7 is consistent with the higher tricyclic component. (*G*–*K*) CYP1A immunofluorescence (epifluorescent images) of representative OGE-exposed embryos from experiments shown in (*F*) and similar experiments with AhR2 single morphants, fixed at 42 hpf. (*G*) Uninjected control with intense epidermal CYP1A signal. (*H*) AhR2-MIS–injected control. (*I*) AhR2 morphant with loss of epidermal CYP1A signal and robust vascular immunofluorescence. (*J*) AhR1/AhR2 double morphant with markedly reduced overall immunofluorescence. (*K*) CYP1A morphant with background staining only. Bars= 100 μm (*A, B*), 50 μm (*C*–*E*), and 500 μm (*G*–*K*).

Embryos exposed to WAF or OGE showed identical robust patterns of CYP1A induction by 36 hpf. In OGE-exposed embryos, CYP1A immunofluorescence (Figure 5, green) was strong in the epidermis (Figure 5B), cranial vasculature (Figure 5C), trunk vasculature (data not shown), and the endothelium lining both the atrium (Figure 5D) and ventricle (Figure 5E), but was absent in the myocardium (Figure 5D,E, red fluorescence). CGE-exposed embryos showed only background immunofluorescence (Figure 5A). As we observed for chrysene, injection of AhR2-MO blocked the epidermal induction of CYP1A by weathered crude oil, leaving the vascular induction intact (Figure 5I). Confocal imaging showed that the vascular CYP1A immunofluorescence was actually higher in AhR2 morphants than in uninjected or control morphants (data not shown). Nevertheless, AhR2-MO injection, in three separate experiments with maximum morpholino levels allowing viability, did not influence the cardiac dysfunction or intracranial hemorrhage caused by weathered crude oil exposure (*n* > 200, data not shown). In contrast, coinjection of AhR1-MO and AhR2-MO markedly reduced CYP1A immunofluorescence in both the epidermis and vasculature in OGE-exposed embryos (Figure 5J). CYP1A immunofluorescence was virtually eliminated in OGE-exposed CYP1A morphants (Figure 5K). In contrast to what we observed with pyrene, defects were more severe in OGE-exposed morphants with an inactive AhR pathway (Figure 5F). At the earliest time point assayed (39 hpf), both AhR1/AhR2 double morphants and CYP1A morphants showed a significantly larger fraction with no circulating erythrocytes due to severe reductions in contractility (~ 50%, chi-square *p* < 0.001), and weak circulation with atrial regurgitation (~150% of controls, chi-square *p* < 0.001). Although a very small but statistically significant increase in cardiac dysfunction was observed in either control or antisense morpholino-injected embryos incubated in CGE, the nature of the cardiac defects was different from that seen with weathered crude oil exposure and consistent with injection artifact (data not shown). Overall, these data indicate that the AhR pathway actually provides some degree of protection from PAH-induced cardiac toxicity.

Because cardiac output and intracranial hemorrhage are likely to be functionally related, it was difficult to determine whether AhR activation or CYP1A induction played a causal role in intracranial hemorrhage. Both CYP1A morphants and AhR1/AhR2 double morphants had a reduced overall occurrence of intracranial hemorrhage, but this was most likely due to the preponderance of embryos with no circulation. In these animals, erythrocytes would pool in the yolk sac. Intracranial hemorrhage was still present among most embryos that had circulation and a lack of CYP1A immunofluorescence (data not shown), which strongly suggests that the underlying pathophysiology is AhR independent. Because morpholinos are most effective at earlier developmental stages, we focused primarily on cardiac function. We did not assess whether the finfold defects resulting from weathered crude oil exposure required a functional AhR pathway.

## Discussion

PAHs of different molecular weight and structure generally are thought to share a common toxic mechanism mediated by the AhR pathway. The data presented here demonstrate that different PAH classes act via distinct toxic mechanisms during fish development. Moreover, our data reveal the complexity of different PAH classes with respect to their distribution in tissues and interactions with AhR family members. The very low abundance of zebrafish AhR1 mRNA coupled with a lack of TCDD binding and transactivation ability suggested that it encodes a nonclassical receptor (Andreasen et al. 2002). However, non-halogenated PAHs were not tested in those studies, and it is clear from our results that AhR1 can be activated by some ligands. Although generally nontoxic to zebrafish embryos, chrysene appears to activate AhR1 and AhR2 *in vivo* in different tissues. Although the tricyclic PAHs and pyrene cause primarily vascular endothelial CYP1A induction, exposure to chrysene results in extensive CYP1A expression in the epidermis, as well. Although either AhR1 or AhR2 can mediate the vascular induction of CYP1A by chrysene, the epidermal induction of CYP1A by chrysene is AhR2 specific. The simplest interpretation is that chrysene accumulates in or binds to epidermal cells, possibly due to its higher hydrophobicity, whereas pyrene and the tricyclic PAHs do not, and that epidermal cells do not express AhR1. In contrast, based on the present results, we would predict that phenanthrene, dibenzothiophene, and pyrene selectively bind zebrafish AhR2 over AhR1.

Of the four model PAHs examined here, only one—pyrene—has dioxin-like (i.e., AhR-dependent) toxicity. Although there is considerable overlap between the effects of pyrene and TCDD on zebrafish larvae, there are distinct features to both syndromes. For example, exposure to pyrene during embryogenesis does not result in the jaw malformations observed with TCDD exposure. Similarly, although there is increased apoptotic cell death in the brain of TCDD-exposed larvae (Dong et al. 2002), the developing spinal cord does not show the widespread cell death as observed with pyrene exposure. Most important, although CYP1A knockdown failed to modulate TCDD toxicity (Carney et al. 2004), the onset of pyrene toxicity was markedly delayed in CYP1A morphants. Most evidence suggests that TCDD toxicity is related to its poor metabolism by CYP enzymes and concomitant bioaccumulation with persistent, futile AhR activation. Moreover, the toxicity of halogenated aromatic compounds generally correlates with affinity for the AhR (Hestermann et al. 2000) and, by extension, the degree of CYP1A induction. Of the model PAHs tested here, chrysene is the most potent CYP1A inducer [two orders of magnitude greater than pyrene (Barron et al. 2004)] but the least toxic. Thus, even though metabolites of some individual PAHs (e.g., pyrene) may be embryotoxic, CYP1A induction or catalytic activity in itself appears to play little role in the developmental toxicity of petrogenic PAHs. Similarly, because chrysene had no overt toxicity in AhR1/AhR2 double or CYP1A morphants, neither unmetabolized chrysene nor CYP1A-derived metabolites are likely to contribute to the embryotoxicity of weathered crude oil.

Compared with urbanized aquatic systems, where PAHs are almost always mixed with other contaminants (e.g., metals, persistent organochlorines, pesticides), the weathered ANS crude oil model provides a fairly simple system for dissecting the effects of an environmentally relevant PAH mixture. The effects of weathered crude oil exposure have little in common with pyrene or TCDD toxicity and instead overlap to a surprising degree with those of individual tricyclic PAHs. The earliest and most pronounced effect of weathered crude oil is on cardiac function during looping stages and subsequent cardiac morphogenesis. These findings, coupled with our previous work (Incardona et al. 2004), indicate that the tricyclic components of weathered crude oil are the major toxic fraction and that the abundant alkylated PAHs probably have cardiac-specific activities similar to the nonalkylated homologs. Moreover, toxicity can be attributed to the parent compounds because metabolism of PAHs via the AhR pathway confers a degree of protection. These findings have widespread implications for ecological assessment and natural resource management.

First, PAH-contaminated environments are often characterized by mixtures derived from both petrogenic (e.g., oil spills) and pyrogenic (e.g., vehicle exhaust) sources. Within a mixture, the composition of individual PAHs can vary considerably from low- to high-molecular-weight compounds. Because different PAHs act on fish embryos via independent toxic mechanisms, understanding the cumulative toxicity of PAH mixtures will be more challenging than previously appreciated.

Second, these findings implicate cardiovascular dysfunction as a major proximal cause of deformities associated with exposure to petrogenic PAHs. This raises the possibility that lower exposure concentrations cause subtle cardiovascular effects in fish that otherwise appear normal. This might explain, for example, the reduced marine survival of pink salmon exposed as embryos to lower levels of weathered ANS crude oil (Heintz et al. 2000).

Third, the variety of cardiac function abnormalities we observed with both model tricyclic PAHs and weathered crude oil suggests several potential targets in the myocardium, which, in addition to HERG potassium channels, includes sarcoplasmic or plasma membrane calcium channels, as well as gap junctions. In this light, it is notable that among PAHs with two to five rings, tricyclic compounds were the most potent in blocking dye coupling via gap junctions in an *in vitro* assay (Blaha et al. 2002). The identification of direct cardiovascular pathophysiology as a key process underlying petrogenic PAH toxicity provides a conceptual framework for the development of better tools for assessing PAH effects in wild freshwater and marine fish.

Fourth, our findings emphasize the current limitations to assessing the effects of oil spills and other sources of aquatic PAH contamination. For many years, measuring CYP1A in field-collected samples has been the basis for assessing ecological damage and recovery after oil spills or remediation efforts in urbanized watersheds. However, CYP1A appears to play a protective rather than a causal role in petrogenic PAH toxicity. This greatly reduces the significance of CYP1A as a biomarker of PAH effect.

Finally, these results highlight the relative toxicity of low-molecular-weight tricyclic PAHs, which previously were considered to be weakly toxic based on the AhR agonist model. Phenanthrene inputs into the environment have remained constant in the last two decades, even in areas receiving inputs of predominantly pyrogenic PAHs (Lima et al. 2002). An understanding of the impacts of tricyclic PAHs on at-risk fish species thus deserves greater emphasis.

## Supplementary Material

Supplemental Table 1

Supplemental Movie 1

Supplemental Movie 2

Supplemental Movie 3

Supplemental Movie 4

## Figures and Tables

**Table 1:** AhR2 morpholino prevents pyrene toxicity but not tricyclic PAH toxicity (%).

Treatment	CYP1A^+^ at 48 hpf	Cardiac dysfunction at 48 hpf	Viability at 106 hpf
AhR2-MIS + 28 μM phenanthrene	100 (*n* = 17)	100 (*n* = 22)	—
AhR2-MO + 28 μM phenanthrene	7 (*n* = 27)	100 (*n* = 72)	—
Uninjected + 28 μM dibenzothiophene	100 (*n* = 20)	100 (*n* = 20)	—
AhR2-MO + 28 μM dibenzothiophene	0 (*n* = 17)	100 (*n* = 17)	—
Uninjected + DMSO	0 (*n* = 43)	—	100 (*n* = 90)
Uninjected + 5 μM pyrene	100 (*n* = 39)	—	9 (*n* = 93)
AhR2-MIS + 5 μM pyrene	94 (*n* = 16)	—	0 (*n* = 70)
AhR2-MO + 5 μM pyrene	3 (*n* = 31)	—	86 (*n* = 56)
CYP1A-MO + 5 μM pyrene	7 (*n* = 59)	—	92 (*n* = 39)^a^

—, not applicable.

a Mortality delayed 18 hr relative to control; 0% viable at 124 hpf.

**Table 2:** Summary of PAH levels in weathered crude oil exposures.

Weathered oil preparation (experiment)	Total PAH (μg/L)	Tricyclic PAHs (%)
WAF day 1	1,549	43.7
WAF day 4	264	55.8
OGE day 1 (column 1)	78.0	16.9
OGE day 4 (column 1)	53.5	24.1
OGE day 0 (column 6)	111.1	17.6
OGE day 2 (column 6)	53.2	25.8
OGE day 7 (column 6)	52.7	28.9

## References

[b1-ehp0113-001755] Andreasen EA, Hahn ME, Heideman W, Peterson RE, Tanguay RL (2002). The zebrafish (*Danio rerio*) aryl hydrocarbon receptor type 1 is a novel vertebrate receptor. Mol Pharmacol.

[b2-ehp0113-001755] Bader D, Masaki T, Fischman DA (1982). Immunochemical analysis of myosin heavy chain during avian myogenesis *in vivo* and *in vitro*. J Cell Biol.

[b3-ehp0113-001755] Barron MG, Heintz RA, Rice SD (2004). Relative potency of PAHs and heterocycles as aryl hydrocarbon receptor agonists in fish. Mar Environ Res.

[b4-ehp0113-001755] Belair CD, Peterson RE, Heideman W (2001). Disruption of erythropoiesis by dioxin in the zebrafish. Dev Dyn.

[b5-ehp0113-001755] Berdougo E, Coleman H, Lee DH, Stainier DY, Yelon D (2003). Mutation of *weak atrium/atrial myosin heavy chain* disrupts atrial function and influences ventricular morphogenesis in zebrafish. Development.

[b6-ehp0113-001755] Blaha L, Kapplova P, Vondracek J, Upham B, Machala M (2002). Inhibition of gap-junctional intercellular communication by environmentally occurring polycyclic aromatic hydrocarbons. Toxicol Sci.

[b7-ehp0113-001755] Carls MG, Rice SD, Hose JE (1999). Sensitivity of fish embryos to weathered crude oil: Part I. Low-level exposure during incubation causes malformations, genetic damage, and mortality in larval Pacific herring (*Clupea pallasi*). Environ Toxicol Chem.

[b8-ehp0113-001755] Carney SA, Peterson RE, Heideman W (2004). 2,3,7,8-Tetrachlorodibenzo-*p*-dioxin activation of the aryl hydrocarbon receptor/aryl hydrocarbon receptor nuclear translocator pathway causes developmental toxicity through a CYP1A-independent mechanism in zebrafish. Mol Pharmacol.

[b9-ehp0113-001755] Couillard CM (2002). A microscale test to measure petroleum oil toxicity to mummichog embryos. Environ Toxicol.

[b10-ehp0113-001755] Dong W, Teraoka H, Yamazaki K, Tsukiyama S, Imani S, Imagawa T (2002). 2,3,7,8-Tetrachlorodibenzo-*p*-dioxin toxicity in the zebrafish embryo: local circulation failure in the dorsal midbrain is associated with increased apoptosis. Toxicol Sci.

[b11-ehp0113-001755] Draper BW, Morcos PA, Kimmel CB (2001). Inhibition of zebrafish *fgf8* pre-mRNA splicing with morpholino oligos: a quantifiable method for gene knockdown. Genesis.

[b12-ehp0113-001755] Ensembl Zebrafish Genome Browser 2005. Ensembl Homepage. Available: http://www.ensembl.org/Danio_rerio/index.html [accessed 27 October 2005].

[b13-ehp0113-001755] Fabricius KE (2005). Effects of terrestrial runoff on the ecology of corals and coral reefs: review and synthesis. Mar Pollut Bull.

[b14-ehp0113-001755] GenBank 2005. GenBank Overview. Available: http://www.ncbi.nlm.nih.gov/Genbank/index.html [accessed 27 October 2005].

[b15-ehp0113-001755] Hahn ME (2002). Aryl hydrocarbon receptors: diversity and evolution. Chem Biol Interact.

[b16-ehp0113-001755] Heintz RA, Rice SD, Wertheimer AC, Bradshaw RF, Thrower FP, Joyce JE (2000). Delayed effects on growth and marine survival of pink salmon *Oncorhynchus gorbuscha* after exposure to crude oil during embryonic development. Mar Ecol Prog Ser.

[b17-ehp0113-001755] Heintz RA, Short JW, Rice SD (1999). Sensitivity of fish embryos to weathered crude oil: Part II. Increased mortality of pink salmon (*Oncorhynchus gorbuscha*) embryos incubating downstream from weathered Exxon Valdez crude oil. Environ Toxicol Chem.

[b18-ehp0113-001755] Henry TR, Spitsbergen JM, Hornung MW, Abnet CC, Peterson RE (1997). Early life stage toxicity of 2,3,7,8-tetrachlorodibenzo-*p*-dioxin in zebrafish (*Danio rerio*). Toxicol Appl Pharmacol.

[b19-ehp0113-001755] Hestermann EV, Stegeman JJ, Hahn ME (2000). Relative contributions of affinity and intrinsic efficacy to aryl hydrocarbon receptor ligand potency. Toxicol Appl Pharmacol.

[b20-ehp0113-001755] Incardona JP, Collier TK, Scholz NL (2004). Defects in cardiac function precede morphological abnormalities in fish embryos exposed to polycyclic aromatic hydrocarbons. Toxicol Appl Pharmacol.

[b21-ehp0113-001755] Karchner SI, Powell WH, Hahn ME (1999). Identification and functional characterization of two highly divergent aryl hydrocarbon receptors (AHR1 and AHR2) in the teleost *Fundulus heteroclitus*. Evidence for a novel subfamily of ligand-binding basic helix loop helix-Per-ARNT-Sim (bHLH-PAS) factors. J Biol Chem.

[b22-ehp0113-001755] Kimmel CB, Ballard WW, Kimmel SR, Ullmann B, Schilling TF (1995). Stages of embryonic development of the zebrafish. Dev Dyn.

[b23-ehp0113-001755] Langheinrich U, Vacun G, Wagner T (2003). Zebrafish embryos express an orthologue of HERG and are sensitive toward a range of QT-prolonging drugs inducing severe arrhythmia. Toxicol Appl Pharmacol.

[b24-ehp0113-001755] Li D, Daler D (2004). Ocean pollution from land-based sources: East China Sea, China. Ambio.

[b25-ehp0113-001755] Lima ALC, Eglinton TI, Reddy CM (2002). High-resolution record of pyrogenic polycyclic aromatic hydrocarbon deposition during the 20th century. Environ Sci Technol.

[b26-ehp0113-001755] Marty GD, Short JW, Dambach DM, Willits NH, Heintz RA, Rice SD (1997). Ascites, premature emergence, increased gonadal cell apoptosis, and cytochrome P4501A induction in pink salmon larvae continuously exposed to oil-contaminated gravel during development. Can J Zool-Rev Can Zool.

[b27-ehp0113-001755] National Research Council 2003. Oil in the Sea III: Inputs, Fates, and Effects. Washington, DC:National Academies Press. 25057607

[b28-ehp0113-001755] Nebert DW, Dalton TP, Okey AB, Gonzalez FJ (2004). Role of aryl hydrocarbon receptor-mediated induction of the CYP1 enzymes in environmental toxicity and cancer. J Biol Chem.

[b29-ehp0113-001755] Park SS, Miller H, Klotz AV, Kloepper-Sams PJ, Stegeman JJ, Gelboin HV (1986). Monoclonal antibodies to liver microsomal cytochrome P-450E of the marine fish *Stenotomus chrysops* (scup): cross reactivity with 3-methylcholanthrene induced rat cytochrome P-450. Arch Biochem Biophys.

[b30-ehp0113-001755] Peterson CH, Rice SD, Short JW, Esler D, Bodkin JL, Ballachey BE (2003). Long-term ecosystem response to the Exxon Valdez oil spill. Science.

[b31-ehp0113-001755] Peterson RE, Theobald HM, Kimmel GL (1993). Developmental and reproductive toxicity of dioxins and related compounds: cross-species comparisons. Crit Rev Toxicol.

[b32-ehp0113-001755] Pew Oceans Commission 2003. America’s Living Oceans: Charting a Course for Sea Change. Arlington, VA:Pew Oceans Commission.

[b33-ehp0113-001755] Phillips DH (1983). Fifty years of benzo(*a*)pyrene. Nature.

[b34-ehp0113-001755] Pollino CA, Holdway DA (2002). Toxicity testing of crude oil and related compounds using early life stages of the crimson-spotted rainbowfish (*Melanotaenia fluviatilis*). Ecotox Environ Saf.

[b35-ehp0113-001755] Prasch AL, Teraoka H, Carney SA, Dong W, Hiraga T, Stegeman JJ (2003). Aryl hydrocarbon receptor 2 mediates 2,3,7,8-tetrachlorodibenzo-*p*-dioxin developmental toxicity in zebrafish. Toxicol Sci.

[b36-ehp0113-001755] Reddy CM, Eglinton TI, Hounshell A, White HK, Xu L, Gaines RB (2002). The West Falmouth oil spill after thirty years: the persistence of petroleum hydrocarbons in marsh sediments. Environ Sci Technol.

[b37-ehp0113-001755] Schmidt JV, Bradfield CA (1996). Ah receptor signaling pathways. Annu Rev Cell Dev Biol.

[b38-ehp0113-001755] Short JW, Heintz RA (1997). Identification of *Exxon Valdez* oil in sediments and tissues from Prince William Sound and the northwestern Gulf of Alaska based on a PAH weathering model. Environ Sci Technol.

[b39-ehp0113-001755] Short JW, Lindeberg MR, Harris PM, Maselko JM, Pella JJ, Rice SD (2004). Estimate of oil persisting on the beaches of Prince William Sound 12 years after the Exxon Valdez oil spill. Environ Sci Technol.

[b40-ehp0113-001755] Sloan CA Brown DW Pearce RW Boyer RH Bolton JL Burrows DG 2004. Extraction, Cleanup and Gas Chromatography/Mass Spectrometry Analysis of Sediments and Tissues for Organic Contaminants. NMFS-NWFSC-59. Seattle, WA:U.S. Department of Commerce, National Oceanic and Atmospheric Administration, National Marine Fisheries Service.

[b41-ehp0113-001755] Teraoka H, Dong W, Tsujimoto Y, Iwasa H, Endoh D, Ueno N (2003). Induction of cytochrome P450 1A is required for circulation failure and edema by 2,3,7,8-tetrachlorodibenzo-*p*-dioxin in zebrafish. Biochem Biophys Res Commun.

[b42-ehp0113-001755] U.S. Commission on Ocean Policy 2004. An Ocean Blueprint for the 21st Century: Final Report of the U.S. Commission on Ocean Policy—Pre-Publication Copy. Washington, DC:U.S. Commission on Ocean Policy. Available: http://www.oceancommission.gov/documents/full_color_rpt/wel-come.html [accessed 26 October 2005].

[b43-ehp0113-001755] Van Metre PC, Mahler BJ (2003). The contribution of particles washed from rooftops to contaminant loading to urban streams. Chemosphere.

[b44-ehp0113-001755] Van Metre PC, Mahler BJ, Furlong ET (2000). Urban sprawl leaves its PAH signature. Environ Sci Technol.

[b45-ehp0113-001755] Whyte JJ, Jung RE, Schmitt CJ, Tillitt DE (2000). Ethoxyresorufin-*O*-deethylase (EROD) activity in fish as a biomarker of chemical exposure. Crit Rev Toxicol.

