# Supplementary material for: Aryl Hydrocarbon Receptor–Independent Toxicity of Weathered Crude Oil during Fish Development
Source: Environ Health Perspect. 2005 Aug 10;113(12):1755–62. doi: 10.1289/ehp.8230 (PMC1315066; doi:10.1289/ehp.8230)

Incardona et al., Aryl Hydrocarbon Receptor-Independent Toxicity Of Weathered Crude Oil During Fish Development

**Supplemental Material**

**Supplemental Table 1: Flow rates and temperatures for oiled gravel column experiments.**

| Experiment | column flow rate: mean, min, max (l/hr) | effluent temp: mean, min, max (˚C) |
|---|---|---|
| 1 control column | 0.49, 0.12, 1.04 | 21.6, 21.0, 23.5 |
| oiled column | 0.55, 0.29, 1.13 | 21.7, 20.5, 23.0 |
| 2 control column | 1.08, 0.24, 1.98 | 24.0, 23.5, 24.5 |
| oiled column | 1.12, 0.30, 1.86 | 24.2, 24.0, 24.5 |
| 3 control column | 1.29, 0.33, 3.36 | 24.9, 24.0, 25.5 |
| oiled column | 1.45, 0.36, 3.36 | 25.0, 24.5, 25.5 |
| 4 control column | 0.85, 0.36, 1.32 | 24.7, 24.0, 25.0 |
| oiled column | 0.90, 0.30, 2.16 | 24.7, 24.0, 25.0 |
| 5 control column | 0.78, 0.21, 1.92 | 24.7, 24.0, 26.0 |
| oiled column | 0.84, 0.33, 1.74 | 25.0, 24.0, 25.5 |
| 6 control column | 1.14, 0.24, 2.40 | 24.8, 24.0, 25.5 |
| oiled column | 1.14, 0.24, 2.52 | 25.2, 25.0, 26.0 |

**Supplemental Table 2: Abbreviations of PAH analytes shown in Supplemental Figures 2 and 3**

| PAH | Abbreviation |
|---|---|
| Naphthalene | NPH |
| Biphenyl | BPH |
| C-1 naphthalenes | C1NPH |
| C-2 naphthalenes | C2NPH |
| C-3 naphthalenes | C3NPH |
| C-4 naphthalenes | C4NPH |
| Acenaphthylene | ACY |
| Acenaphthene | ACE |
| Fluorene | FLU |
| C-1 fluorenes | C1FLU |
| C-2 fluorenes | C2FLU |
| C-3 fluorenes | C3FLU |
| Dibenzothiophene | DBT |
| C-1 dibenzothiophenes | C1DBT |
| C-2 dibenzothiophenes | C2DBT |
| C-3 dibenzothiophenes | C3DBT |
| Phenanthrene | PHN |
| Anthracene | ANT |

| | |
|---|---|
| C-1 phenanthrenes | C1PHN |
| C-2 phenanthrenes | C2PHN |
| C-3 phenanthrenes | C3PHN |
| C-4 phenanthrenes | C4PHN |
| Fluoranthene | FLU |
| Pyrene | PYR |
| C-1 fluoranthenes | C1FLA |
| Benz[a]anthracene | BAA |
| Chrysene | CHR |
| C-1 chrysenes | C1CHR |
| C-2 chrysenes | C2CHR |
| C-3 chrysenes | C3CHR |
| C-4 chrysenes | C4CHR |
| Benzo-b-fluoranthene | BbF |
| Benzo-k-fluoranthene | BkF |
| Benzo-e-pyrene | BEP |
| Benzo-a-pyrene | BAP |
| Indeno-123-cd-pyrene | IDP |
| Dibenzo-a,h-anthracene | DBA |
| Benzo-g,h,i-perylene | BZP |

**Supplemental Figure Legends:**

Supplemental Figure 1: (A) Structures of PAH compounds discussed in the text. (B) Structures of zebrafish AhRs. The AhR1 transcript structure is shown at top, with the predicted exon skipping caused by I2E3 morpholino in red. Protein domain structures for AhR1 and AhR2 are compared below, with the predicted truncated AhR1 form encoded by the transcript with exon 3 deleted by I2E3 morpholino. Protein domains are *bHLH*, basic helix-loop-helix; *PAS*, Per-Arnt-Sim (A and B); and *LBD*, ligand-binding domain. The LBD encompasses the PAS-B domain, and the predicted shifted open reading frame with premature termination in exon 4 is indicated in red in AhR1-I2E3.

Supplemental Figure 2: Representative PAH composition of water from zebrafish weathered crude oil exposures. Composition is shown for (top to bottom) control gravel effluent, oiled gravel effluent on days 1 and 4 (experiment 1, Supplemental Table 1), and water-accomodated fraction (*WAF*) on days 1 and 4. Exposures started on day 0; embryos were ≥24hpf on day 1. Total PAH levels are indicated for each set, as are the percentages for the sum of the most abundant homologous chemical groups (left to right): naphthalenes, fluorenes, dibenzothiophenes, phenanthrenes, and chrysenes. Abbreviations for all PAH analytes are provided in Supplemental Table 2.

Supplemental Figure 3: PAH composition of exposure water from experiment represented by main text Figure 5. Composition is shown for (top to bottom) control gravel effluent, and oiled gravel effluent on days 0, 2, 4, and 7 (experiment 6, Supplemental Table 1). Total PAH levels are

indicated for each set, as are the percentages for the sum of the most abundant homologous chemical groups (left to right): naphthalenes, fluorenes, dibenzothiophenes, phenanthrenes, and chrysenes. Abbreviations for all PAH analytes are provided in Supplemental Table 2. Column flow was stopped overnight between days 4 and 5. Total PAH levels were higher on day 7 than day 4 most likely due to equilibration of the effluent during stasis of flow.

**Supplemental Movie Legends:**

Supplemental Movie 1: Phenanthrene or dibenzothiophene causes higher degree AV block in AhR2 morphants. The movie shows a sequence of six high magnification lateral views of the beating heart at 48 hpf with anterior to the left and dorsal at top. The *eye* and *yolk* are labeled as such, and arrowheads indicate the atrium (*A*) and ventricle (*V*). (1) Normal cardiac rhythm in an uninjected embryo exposed to solvent (DMSO). (2) Partial AV block with one ventricular beat for every two atrial beats in an uninjected embryo exposed to 28 $\mu$M phenanthrene. (3) AhR2 morphant exposed to 28 $\mu$M phenanthrene with higher degree AV block (as many as 4-7 atrial beats to one ventricular beat). (4) AhR2 morphant exposed to 28 $\mu$M phenanthrene with higher degree AV block (as many as 4-7 atrial beats to one ventricular beat). (5) Partial AV block with one ventricular beat for every two atrial beats in an uninjected embryo exposed to 27 $\mu$M dibenzothiophene. (6) AhR2 morphant exposed to 27 $\mu$M dibenzothiophene with higher degree AV block (as many as 4-5 atrial beats to one ventricular beat).

Supplemental Movie 2: Exposure to weathered crude oil causes early cardiac dysfunction and abnormal looping. Embryos incubated in control gravel effluent (CGE-exposed, left) or oiled gravel effluent (OGE-exposed, right) are shown at 36 hpf (top) and 64 hpf (bottom). Anterior is

to the left, dorsal is at top, and the arrowheads indicate the atria (*A*) and ventricles (*V*). The 64 hpf views are higher magnification. At 36 hpf the OGE-exposed embryo shows pooling of erythrocytes in the yolk sac with weak atrial contractility and a stiff, hypokinetic ventricle. The stretched appearance of the cardiac chambers in the OGE-exposed larva at 64 hpf is indicative of abnormal cardiac looping.

Supplemental Movie 3: Range of early cardiac dysfunction in OGE-exposed embryos. A sequence of 4 ventral views of 39 hpf embryos in their chorions are shown, with anterior to the left or upper left corner and arrowheads indicating the atrium (*A*) and ventricle (*V*). (1) Normal circulation in a CGE-exposed embryo. Normal cardiac contractility results in constant streaming of erythrocytes over the yolk sac with continuous atrial filling. (2) Good circulation in an OGE-exposed embryo. (3) Weak circulation in an OGE-exposed embryo. Atrial regurgitation is evident from the discontinuous streaming of erythrocytes across the yolk. (4) OGE-exposed embryos with poor contractility resulting in no circulation of erythrocytes.

Supplemental Movie 4: Atypical movement in the bulbus arteriosus with exposure to OGE. High magnification lateral views are shown in sequence for a CGE- and OGE-exposed embryo at 42 hpf. Anterior is to the left, and arrowheads indicate the ventricle (*V*) and bulbus arteriosus (*BA*). Because the bulbus arteriosus functions as an elastic chamber that moderates flow from the ventricle, altered movement in its walls probably reflects abnormal ventricular pressures.

Supplemental Figure 1

A

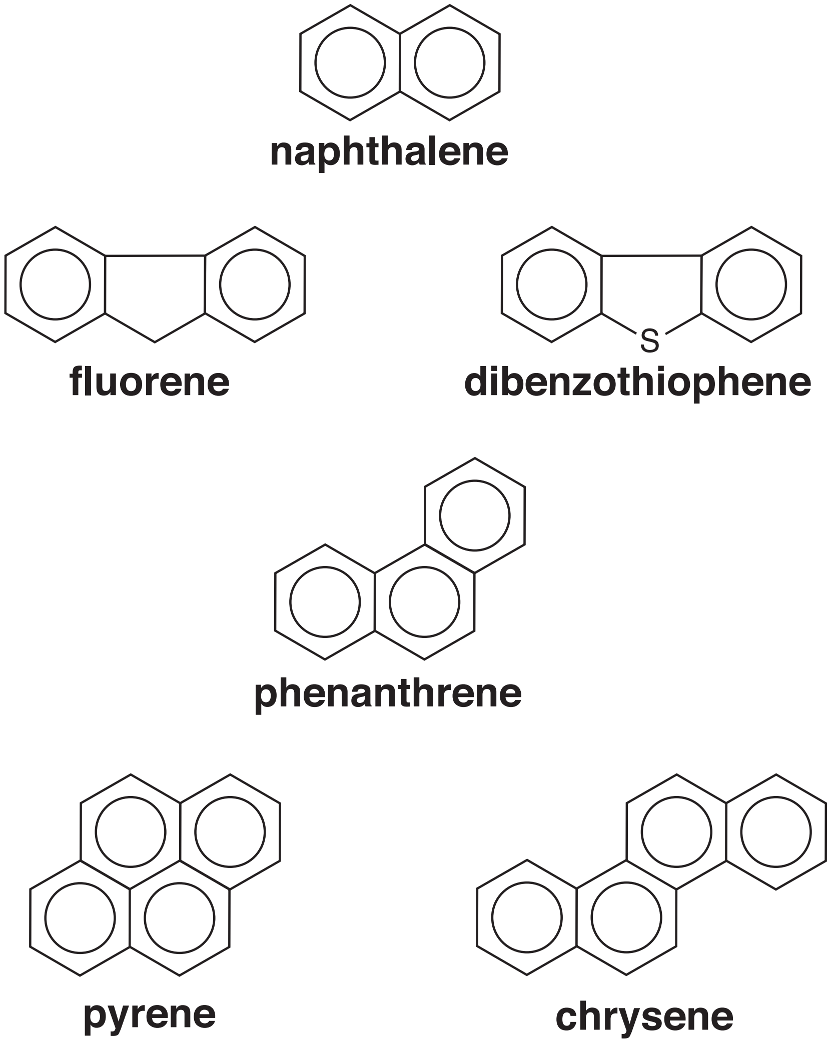


B

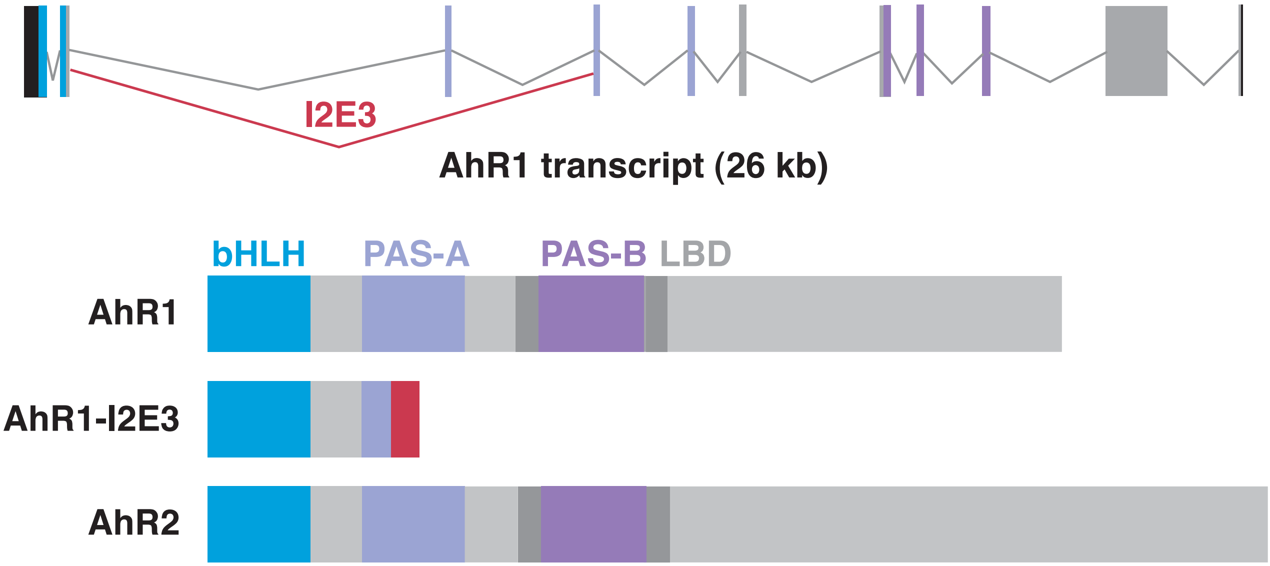

Supplemental Figure 2

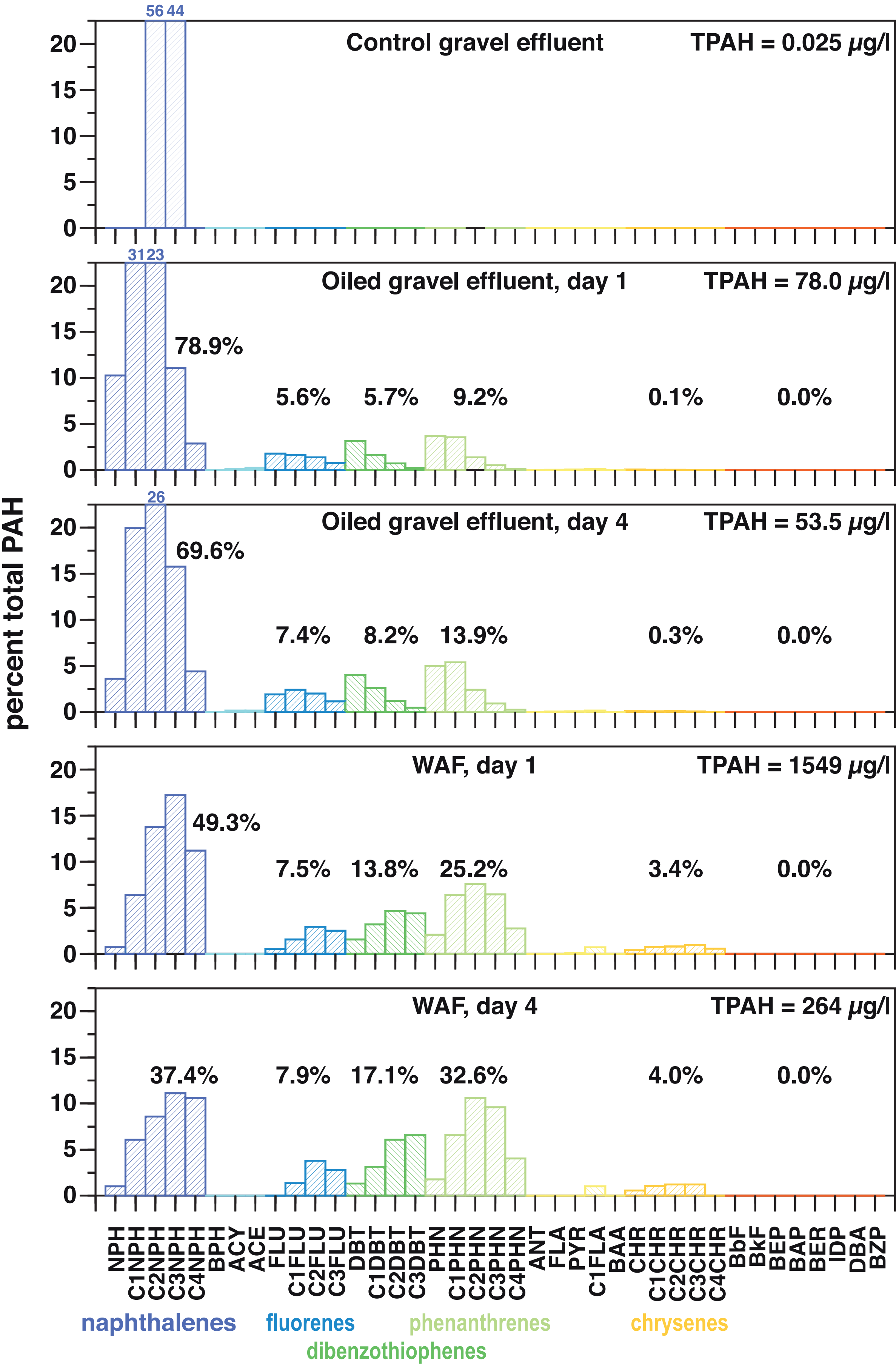

Supplemental Figure 3

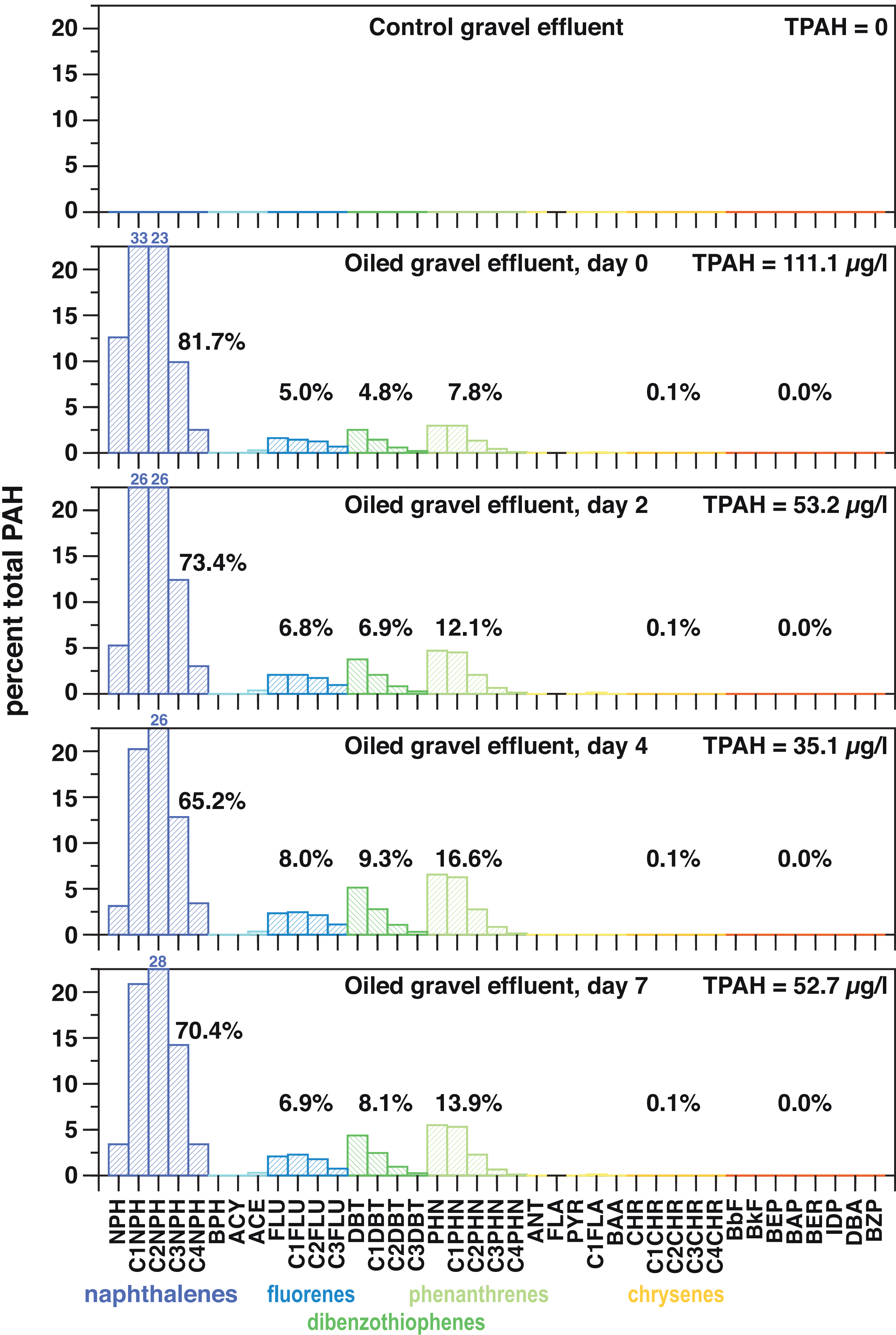

Supplement: Supplemental Table 1 [file ehp0113-001755s1.pdf]
